# Identification of a novel *SGCA* missense mutation in a case of limb‐girdle muscular dystrophy 2D with the absence of four sarcoglycan proteins

**DOI:** 10.1111/neup.12549

**Published:** 2019-04-15

**Authors:** Yanpeng Lu, Xueqin Song, Guang Ji, Hongran Wu, Duan Li, Shuyan Sun

**Affiliations:** ^1^ Department of Neurology The Second Hospital of Hebei Medical University Hebei China

**Keywords:** LGMD2D, next generation sequencing, novel mutation, sarcoglycan, *SGCA*

## Abstract

Limb‐girdle muscular dystrophy 2D (LGMD2D) is caused by mutations in the α‐sarcoglycan gene (*SGCA*). Due to lack of specificity, it is impossible to identify LGMD2D only by clinical symptoms and conventional immunohistochemical staining. The loss of any protein (α‐, β‐, γ‐, δ‐sarcoglycan) that represent sarcoglycanopathy may cause reduction or absence of the other three proteins. Here, we report a patient with a complete loss of all the four proteins. Next generation sequencing (NGS) results showed a missense mutation (C.218 C > T) and a partial heterozygous deletion containing exons 7 and 8 of *SGCA*, which led to the final diagnosis of the patient. The discovery of this new mutation could broaden the spectrum of *SGCA* mutations, which may be associated with putative LGMD2D, especially when all the four proteins are completely missing.

## INTRODUCTION

Limb‐girdle muscular dystrophy 2D (LGMD2D) is a rare autosomal‐recessive myopathy, caused by mutations in the α‐sarcoglycan (SGCA) gene (*SGCA*). Nowadays more than 50 different *SGCA* missense mutations have been reported.^1^ LGMD2D has a significant clinical and genetic heterogeneity, with a broad spectrum of clinical symptoms, mainly characterized by weakness and atrophy of the limb muscles and pelvic muscles with variable age at onset and severity. Even if the same gene mutation occurs in the same family, clinical symptoms range from mild to severe.^2^ In some reports, some patients suffer from exercise intolerance and rhabdomyolysis^3^ or only present with exercise‐induced myalgia and myoglobinuria as first symptoms.^4^ Serum creatine kinase levels are significantly high in these patients. Electromyography (EMG) and muscle biopsy usually suggest myogenic damage. Muscle biopsies are useful for histopathological and immunolabeling studies, and DNA analysis is the gold standard for the definite diagnosis of the specific form of muscular dystrophy.^5^ *SCGA*, which is located on chromosome 17q21 and encompasses 10 exons,^6^ could encode a 387 amino acid protein. To date, over 78 *SGCA* variants have been recognized in humans according to the Human Genome Mutation Database. The most frequently reported mutation, in some populations up to one‐third of *SGCA* mutations, is a missense mutation at position 229 CGC ≥ TGC in exon 3 of *SGCA*, substituting arginine for cysteine in codon 77 (R77C). This is the most frequently reported.^7^ In this report, we applied next generation sequencing (NGS) and quantitative polymerase chain reaction (qPCR) analyses for an accurate classification of muscular dystrophy in a suspected patient whose sarcoglycan proteins were completely absent.

## MATERIALS AND METHODS

### Case report

An 8‐year‐old Chinese boy had problems climbing stairs and slower activity than other peers as noticed 1 year ago. His parents did not notice the boy's behavior when he experienced modest limb pain after a period of activity. Six months ago, the family members noticed that the boy experienced more difficulty in climbing stairs and had to stand up when crouching. Meanwhile, exercise‐induced myalgias became more severe. Then he was admitted to a local hospital, and thoracic and lumbar examinations were performed. However, the results of double‐hip magnetic resonance imaging (MRI) were normal. Thereafter, the boy was admitted to our hospital for further treatment. Neurological examination showed weakened bilateral knee tendon reflexes and hypertrophic bilateral gastrocnemius muscle. Limb strength was assessed at grade 4 and the Gower's sign was positive. The autoantibodies were normal. Biochemical items displayed myocardial enzymes as follows: myoblobulin 448.6 ng/mL, creatine kinase (CK) 14450 U/L, creatine kinase MB fraction 276 U/L, lactic dehydrogenase 961 U/L and normal electrolytes. The family members denied any relevant family history.

### Muscle biopsy

A fresh muscle sample was taken from the patient's left quadriceps and immediately frozen in isopentane cooled by liquid nitrogen. Then the specimens were analyzed using the standard panel of histochemical stains including hematoxylin and eosin (HE), modified Gomori trichrome (MGT), nicotinamide adenine dinucleotide‐tetrazolium reductase (NADH‐TR) and acid phosphatase, adenosine triphosphatase (ATPase) stains, periodic acid‐Schiff (PAS) and oil red O (ORO).^7^ Immunohistochemical staining was carried out using monoclonal antibodies to dysferlin, dystrophin (‐N, ‐C, ‐R), sarcoglycan (α‐, β‐, γ‐, δ‐) as primary antibodies, and horseradish peroxidase‐tagged secondary antibody. A normal muscle sample was selected as healthy control.

### NGS and qPCR analyses

DNA (5 μg) extracted from the patient's muscle was interrupted and amplified. Four hundred and thirty genes associated with muscular dystrophy were captured by using liquid catch kit (Mygenostics, Beijing, China) and sequenced by a high‐throughput sequencing instrument Hisep2000 (Illumina, San Diego, CA, USA), which generated a set of data with mean depth of 414.1‐fold and had a 99.0% coverage of the target sequence at 200× or greater. Biological information of single nucleotide polymorphisms, insertions and deletions were analyzed. The mutant genes of his parents were verified by Sanger sequencing or qPCR according to the results of exon sequencing. The information of the sequenced target region was sorted and analyzed to find the frequency of the mutation in the 1000‐person genome and dbSNP132 database. The pathogenicity was analyzed using Polyphen‐2 software, GATK.

## RESULT

### Clinical symptoms and diagnosis

An 8‐year‐old boy with clinical manifestations of progressive lower extremity weakness and post‐exercise myalgia without relevant family history was admitted to our hospital. Neurological examination showed bilateral gastrocnemius hypertrophy and proximal lower extremity weakness. Laboratory tests showed significantly increased levels of serum CK. Electromyography showed myogenic damage. Therefore, the patient was diagnosed initially as having muscular dystrophy of uncertain type. However, metabolic myopathy could not be excluded.

### Histological results

**Figure 1:**
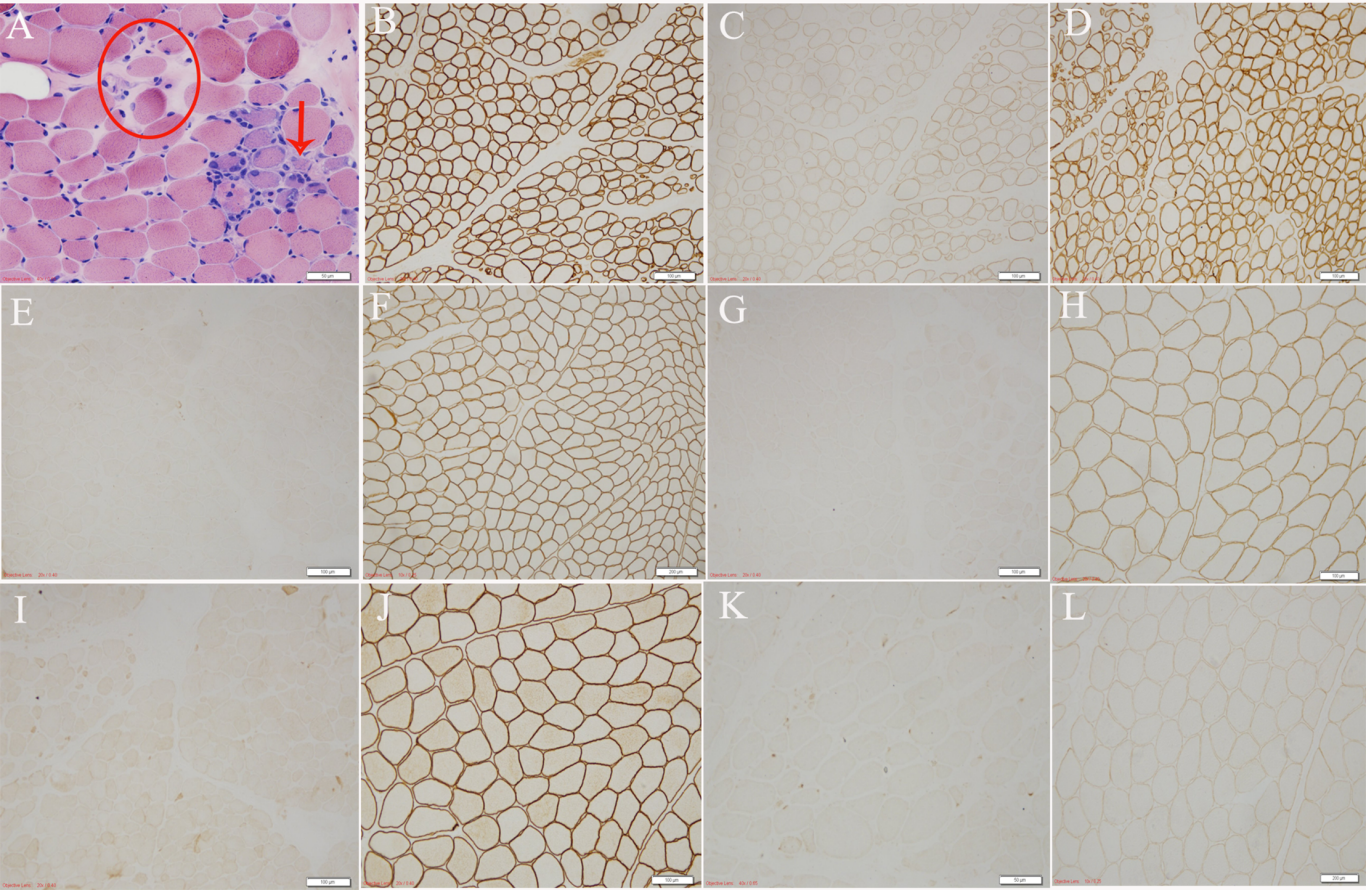
Microphotographs of sections of muscle biopsy specimens from the patient (A‐E, G, I, K) and a normal control (F, H, J, L) stained with HE (A) and immunohistochemically stained for dystrophin (B‐D, N, R) and α‐ (E, F), β‐ (G, H), γ‐ (I, J) and δ‐ (K, L) sarcoglycans. The patient's muscle displays mild hyperplasia of connective and adipose tissues in perimysium and endomysium, which show degeneration, necrosis, phagocytosis and opaque fiber formation as well as loose arrangement of muscle fibers (red circle) and obvious atrophy with a basically circular appearance (arrow) (A). Dystrophic immunoreactivity is normally detected in the patient's muscle fibers (C, N, R). Immunoreactivities for α‐, β‐, γ‐ and δ‐sarcoglycans are detectable in the control's muscle fibers (F, H, J, L) but undetectable in the patient's muscle fibers (E, G, I, K). Scale bars: 50 μm (A, K), 100 μm (B‐E, G‐J), 200 μm (F, L)

HE (Fig. 1A) staining revealed dystrophic changes with muscle fibers loosely arranged in the muscle bundles. The connective tissue and adipose tissue of the muscle were moderately proliferated. No inflammatory cell infiltration was observed. Lipid sedimentary myopathy was excluded because of the absence of lipid droplets on ORO. Immunohistochemical staining (Fig. 1B–L) showed that anti‐α‐, β‐, γ‐, δ‐sarcoglycan proteins were completely absent and anti‐dystrophin‐N, ‐C, ‐R proteins were normal. Thus, Duchenne and Becker muscular dystropies (DMD/BMD) were excluded. Further, NGS was performed for further differential diagnosis toward accurate typing of sarcoglycanopathy.

### Mutation analysis

**Figure 2:**
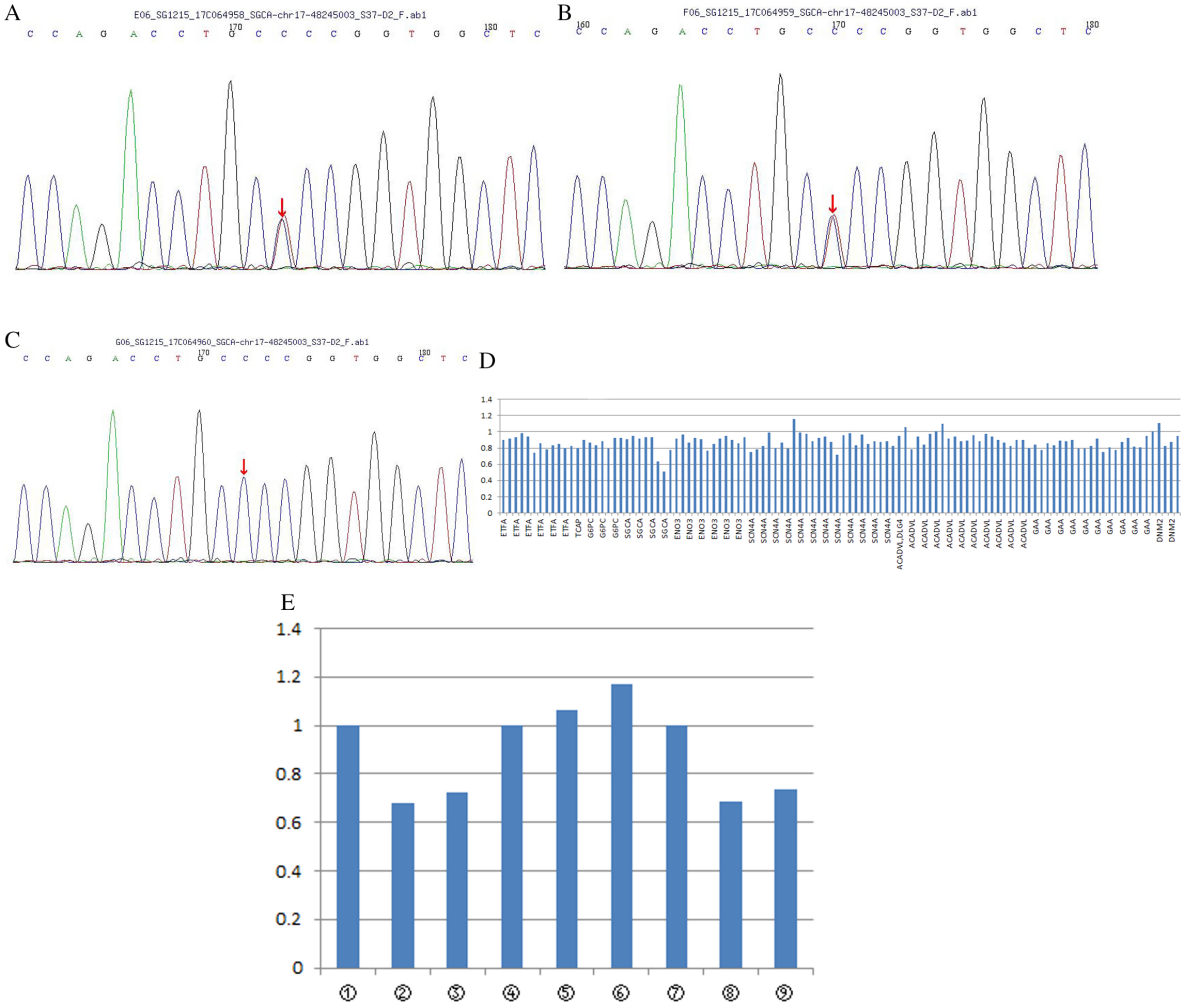
Results of molecular genetic analyses. A missense mutation c.218 C > T (p. Pro 73 Leu) (NM_000023) of *SGCA* (chr17‐48245003) is detected in the patient by NGS (A). The site of the mutation in the patient's father (B) and mother (C) is confirmed by Sanger sequencing. A partial heterozygous deletion is detected in exons 7 and 8 of *SGCA* (D, red circle). A significant reduction in qPCR products for *SGCA* is observed in the patient's (②③) and mother's (⑧⑨) specimens but not the father's (⑤⑥) and control's (①④⑦) specimens.

Two novel heterozygous *SGCA* mutations were revealed. One was missense mutation c.218C > T (cytosine to thymine) leading to amino acid changes p.P73L (proline to leucine), which was further confirmed from the patient's father by Sanger sequencing. Another partial heterozygous deletion contained exons 7–8 and was confirmed by qPCR. This mutation was predicted to be a pathogenic mutation by Polyphen‐2 software (Fig. 2).

## DISCUSSION

Because of locus heterogeneity and low diagnostic specificity in LGMD, there is little information about its prevalence around the world. The prevalence of sarcoglycanopathy varies greatly among different subtypes and ethnic backgrounds. It has been reported that sarcoglycanopathy ranks third among limb‐type muscular dystrophy in Italy.^8^

LGMD2D has a significant genetic and clinical heterogeneity, with a broad spectrum of clinical symptoms. However, almost all symptoms lack specificity. Due to the clinical heterogeneity of LGMD, it is impossible to rely solely on clinical and routine pathological examination for a differential diagnosis. For example, LGMD2D is often accompanied by gastrocnemius hypertrophy^9^ and is not easily differentiated from DMD/BMD.

Routine histologic staining is in favor of muscular dystrophy, but it cannot differentiate sarcoglycanopathy from DMD/BMD. Therefore, further immunohistochemistry was implemented which resulted in the absence of four sarcoglycan proteins, was consistent with previous literature.^10^ MRI has shown that the adductor magnus is the most frequently and severely affected muscle in sarcoglycanopathy,^11^ which provides effective help in the positioning of site selection during muscle tissue biopsy.

Results in our study showed complete absence of the α‐, β‐, γ‐, δ‐sarcoglycan proteins, and normal dystrophin (‐N, ‐C, ‐R), thus DMD/BMD was excluded. Further, LGMD2D was further confirmed by NGS. In LGMD2D patients, the defect of α‐sarcoglycan protein could cause the secondary reduction or complete absence of β‐, γ‐, δ‐sarcoglycan proteins. Any one reduction or absence of any single of sarcoglycan protein complex will result in a change in overall function, leading to pathogenic changes.

In this report, we discovered a previously unknown point mutation (c.218 C > T (p.P73L)) in exon 3, adding to the growing spectrum of mutations in the *SGCA*. Throughout the mutations in the *SGCA*, a high relative frequency of mutations was reported in exon 3 (13/26; 50%).^12^ Another complex heterozygosis deletion was not detected at the corresponding gene locus from both parents by Sanger sequencing. The deletion mutation was verified by qPCR from his mother. Severely abnormal messenger RNA transcribed from abnormal DNA led to the weakness or loss of the encoded protein function, and caused the deletion or abnormal localization of the other three proteins. A similar mutation of rare compound heterozygosity for a deletion encompassing exons 7 and 8 and a missense mutation on different alleles was reported by Madiha Trabelsi *et al.* 12 Therefore, the relationship between mutant mode and protein deletion needs further study. Identification of these mutations is meaningful for genetic counseling and prenatal diagnosis.

## AUTHOR CONTRIBUTIONS

Yanpeng Lu, study concept and design, acquisition of data, analysis and interpretation of data; Xueqin Song, study concept and design, critical revision of manuscript for intellectual content; Guang Ji, analysis and interpretation of data; Hongran Wu, performed a muscle biopsy, acquisition and analysis of pathology data; Duan Li, interpretation of data; Shuyan Sun, acquisition of data.

## DISCLOSURE

This research did not receive any specific grant from funding agencies in the public, commercial, or not‐for‐profit sectors.
